# Enhanced Yield of Recombinant Proteins with Site-Specifically Incorporated Unnatural Amino Acids Using a Cell-Free Expression System

**DOI:** 10.1371/journal.pone.0068363

**Published:** 2013-07-02

**Authors:** Sviatlana Smolskaya, Zhiwen Jonathan Zhang, Lital Alfonta

**Affiliations:** 1 Avram and Stella Goldstein-Goren Department of Biotechnology Engineering, Ben-Gurion University of the Negev, Beer-Sheva, Israel; 2 The Ilse Katz Institute for Nanoscale Science and Technology, Ben-Gurion University of the Negev, Beer-Sheva, Israel; 3 Department of Bioengineering, School of Engineering, Santa Clara University, Santa Clara, California, United States of America; Deutsches Krebsforschungszentrum, Germany

## Abstract

Using a commercial protein expression system, we sought the crucial elements and conditions for the expression of proteins with genetically encoded unnatural amino acids. By identifying the most important translational components, we were able to increase suppression efficiency to 55% and to increase mutant protein yields to levels higher than achieved with wild type expression (120%), reaching over 500 µg/mL of translated protein (comprising 25 µg in 50 µL of reaction mixture). To our knowledge, these results are the highest obtained for both *in vivo* and *in vitro* systems. We also demonstrated that efficiency of nonsense suppression depends greatly on the nucleotide following the stop codon. Insights gained in this thorough analysis could prove useful for augmenting *in vivo* expression levels as well.

## Introduction

The methodology based on unnatural amino acids (UAAs) incorporation into desired loci of the protein of interest is widely used for understanding protein structure-function relationships, investigating protein-based biological processes, and generating proteins and organisms with new properties [1]. Over the past two decades, the most established methods to site-specifically incorporate UAA *in vivo* were based on genetic code expansion. This is accomplished by supplying organisms with a non-endogenous aminoacyl-tRNA synthetase/tRNA pair, referred to as an orthogonal pair, that directs site-specific incorporation of UAA in response to a unique codon [2]. The orthogonal aminoacyl-tRNA synthetase (aaRS) aminoacylates a cognate orthogonal tRNA (but no other cellular tRNAs) with UAA. The orthogonal tRNA is a substrate for the orthogonal aaRS but is not aminoacylated by any endogenous aaRS [3]. The orthogonal aaRS/tRNA pair should, however, be compatible with the translational machinery of the host cell. The first orthogonal aaRS/tRNA pair used in *Escherichia coli* originated from the archaeon, *Methanocaldococcus jannaschii*, and was generated from the tyrosyl-tRNA synthetase and its cognate tRNA pair (*Mj*TyrRS/tRNA^Tyr^) [4]. A unique codon is required to specify the UAA, and two main strategies are generally used: nonsense suppression, i.e. UAA incorporation in response to the least used stop codon recognized by a specific suppressor tRNA [5], [6]; and frame-shift suppression based on the application of four- or five-base extended codons and cognate suppressors [7]–[9].

Nowadays, genetic code expansion in *E. coli* using the amber suppression strategy and evolved variants of orthogonal *M. jannaschii* TyrRS/*Mj*tRNA^Tyr^_CUA_ is considered to be the most established and robust methodology to site-specifically incorporate UAAs. However, this methodology has not been reported to achieve high protein yields. Suppression efficiency depends greatly on the degree of orthogonal tRNA compatibility with the translational apparatus of host cell. The reasons for such an incompatibility are low affinity of the elongation factor Tu (EF-Tu) to UAA-charged *Mj*tRNA_CUA_ derived from archeal tRNA^Tyr^ [10] or its inability to recognize and deliver orthogonal tRNA to the ribosomal A-site [11]. On the other hand, suppression efficiency can be interrupted by the release factors, RF1 and RF2, which are responsible for the release of the growing polypeptide chain from the ribosome. RF functionality depends on the particular stop codon encoded in the mRNA sequence, as well as the fourth base following the stop codon [12], [13]. Recently, tremendous efforts were geared towards the production of greater yields of recombinant proteins containing UAAs, including design of specific vectors encoding multiple copies of *Mj*tRNA_CUA_ [14], identification of newly optimized suppressor tRNA_CUA_^opt^ with modified T-stem [15]–[17], selection of the orthogonal ribosome [18], and selection and application of RF1-depleated *E. coli* variants [19], [20]. However, given the *in vivo* nature of the methodology, overall yield of recombinant protein with site-specifically incorporated UAA has never been reported to exceed 50% comparing to wild type.

Additionally, the evolution of cognate aaRS/tRNA pairs and the incorporation of UAAs into proteins in living organisms is not possible for certain amino acids. These include toxic UAAs, amino acids with extreme redox potentials or hydrophilicity that are not able to cross the cell membrane or UAAs which cannot be generated in large amounts, due to their difficult multi-step chemical synthesis. Moreover, site-directed UAAs incorporation into eukaryotic proteins expressed in mammalian cells is problematic, since no efficient system is available yet.

Herein we suggest complementing caveats that exist in the *in vivo* translational approach by using an efficient cell-free system for the different applications of this robust technology. The cell-free protein translation system can be considered as a good alternative for *in vivo* incorporation of UAAs [21], [22] due to several advantages over current *in vivo* processes, such as possibility to direct all of the cellular resources towards the production of a single protein [23]; to control the level of an orthogonal aaRS/tRNA pair and of the UAA employed in protein expression due to the absence of a cell wall. Another advantage of the *in vitro* approach is the possible use of aforementioned UAAs which can not be applied *in vivo*.

**Figure 1:**
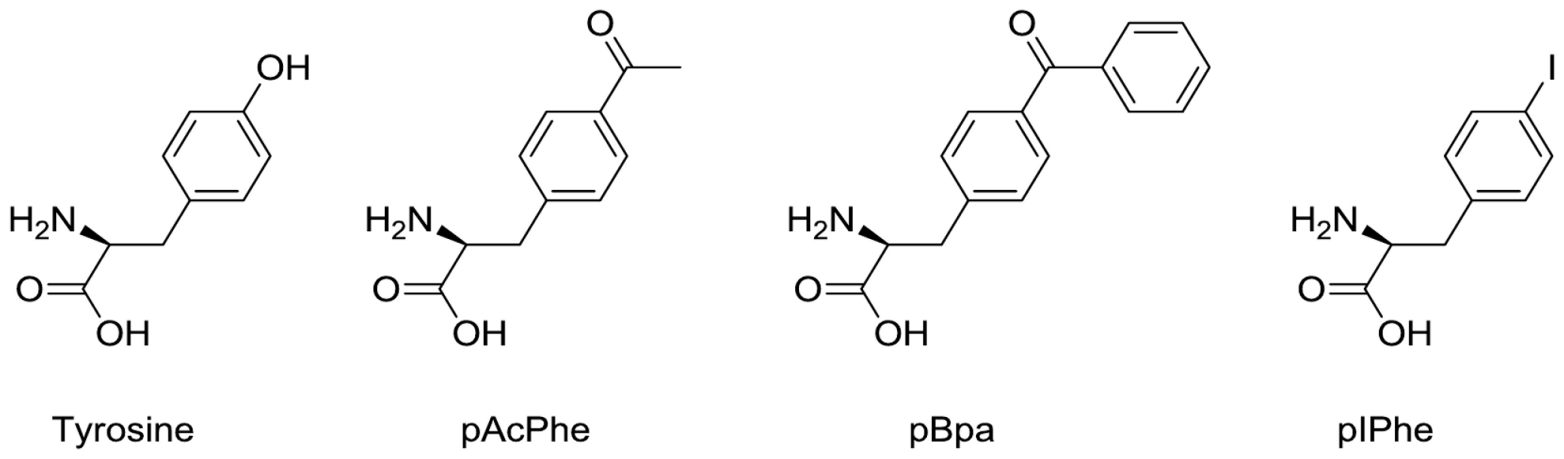
Structures of tyrosine and the three unnatural amino acids used in this study.

Here, we describe a general strategy based on the use of commercially available cell-free expression systems, combined with orthogonal *M. jannaschii* synthetases and cognate *Mj*tRNA_CUA_ or synthetic tRNA_CUA_^Opt^ [4], [24], to obtain high yields of UAA-labeled proteins. This approach allowed us to incorporate tyrosine, as well as p-acetyl-L-phenylalanine (pAcPhe), p-benzoyl-L-phenylalanine (pBpa) and p-Iodo-L-phenylalanine (pIPhe; Fig. 1) into Green Fluorescent Protein (GFP), in response to the TAG stop codon with high fidelity and efficiency. The final yield of modified proteins varied widely and under optimal conditions reached roughly 50–120% of the wild type expression levels, depending on the type of suppressor tRNA, aaRS used and UAA used.

## Materials and Methods

### GFP Mutants

The X-ray crystallographic structure of template GFP (PDB accession number: 1EMA), encoded by the GFP control vector (RTS, 5 PRIME, Hamburg, Germany), was analyzed to choose sites for UAA incorporation. To examine the effect of the nucleotide following the stop codon on protein yields, we selected four amino acid residues on two external β-sheet of GFP and its adjacent loop. The selection of these residues for substitution by an UAA was chosen as not to obstruct proper GFP folding. The coding sequence of GFP was thus modified to replace the codons encoding tyrosine 39, lysine 41, leucine 42 or lysine 45 to an amber stop codon (TAG), with the nucleotide following the stop codon being G, C, A or T, respectively. The codons of amino acids at another β-sheet, i.e. histidine 148, asparagine 149 and valine 150, were substituted to TAG, such that A, G and T followed the stop codon, respectively. In order to generate mutation at permessive site of GFP the codon of tyrosine 151 was replaced to TAG, and the adjacent isoleucine 152 (ATC) was substituted by leucine (CTC), so that C has followed the amber stop codon. Mutagenesis was performed in the control vector containing the gene for GFP, by site-directed mutagenesis using QuikChange II Site-Directed Mutagenesis Kit (Stratagen, Agilent Technologies, Santa Clara, CA) and the following primers:

QC-GFPY39 F (5'–GCATGGCAGGGGTTCAAATCCCCTCCGCCGGA–3') and QC-GFPY39 R (5'–TCCGGCGGAGGGGATTTGAACCCCTGCCATGC–3'); QC-GFPK41 F (5'–GGGTGAAGGTGATGCAACATACGGATAGCTTACCCTTAAATTTATTTGC–3') and QC-GFPK41 R (5'–GCAAATAAATTTAAGGGTAAGCTATCCGTATGTTGCATCACCTTCACCC–3'); QC-GFPL42 F (5'–GATGCAACATACGGAAAATAGACCCTTAAATTTATTTGCAC–3') and QC-GFPL42 R (5'–GTGCAAATAAATTTAAGGGTCTATTTTCCGTATGTTGCATC–3'); QC-GFPK45 F (5'–CATACGGAAAACTTACCCTTTAGTTTATTTGCACTACTGG–3') and QC-GFPK45 R (5'–CCAGTAGTGCAAATAAACTAAAGGGTAAGTTTTCCGTATG–3'); QC-GFPH148 F (5'–GGAATACAACTATAACTCATAGAATGTATACATCATGGCAG–3') and QC-GFPH148 R (5'–CTGCCATGATGTATACATTCTATGAGTTATAGTTGTATTCC–3'); QC-GFPN149 F (5'–CAACTATAACTCACACTAGGTATACATCATGGCAGAC–3') and QC-GFPN149 R (5'–GTCTGCCATGATGTATACCTAGTGTGAGTTATAGTTG–3'); QC-GFPV150 F (5'–GGAATACAACTATAACTCACACAATTAGTACATCATGCAGAC–3') and QC-GFPV150 R (5'–GTCTGCCATGATGTACTAATTGTGTGAGTTATAGTTGTATTCC–3'); QC-GFPY151 F (5'–CTATAACTCACACAATGTATAGCTCATGGCAGACAAACAAAAGAATGG–3') and QC-GFPY151 R (5'–CCATTCTTTTGTTTGTCTGCCATGAGCTATACATTGTGTGAGTTATAG–3'). Simultaneously, lysine 41, leucine 42 or lysine 45 codons were substituted to tyrosine TAC codon applying site-directed mutagenesis methodology and the following primers: QC-K41Y F (5'–GAAGGTGATGCAACATACGGATACCTTACCCTTAAATTTATTTGC–3') and QC-K41Y R (5'–GCAAATAAATTTAAGGGTAAGGTATCCGTATGTTGCATCACCTTC–3'); QC-L42Y F (5'–GGTGATGCAACATACGGAAAATACACCCTTAAATTTATTTGCACTAC–3') and QC-L42Y R (5'–GTAGTGCAAATAAATTTAAGGGTGTATTTTCCGTATGTTGCATCACC–3'); QC-K45Y F (5'–GCAACATACGGAAAACTTACCCTTTACTTTATTTGCACTACTGGAAAAC–3') and QC-K45Y R (5'–GTTTTCCAGTAGTGCAAATAAAGTAAAGGGTAAGTTTTCCGTATGTTGC–3').

### UAA Incorporation Components

The plasmids pSup-*Mj*mutTyrRS-6TRN encoding the orthogonal *M. jannaschii* tyrosyl-synthetase variants (*M. jannaschii* aaRS) for incorporation of tyrosine, pAcPhe and pBpa (Bachem, Bubendorf, Switzerland) and pIPhe (Sigma-Aldrich, St. Louis, MO) and suppressor tRNA were a kind gift from Dr. Peter Schultz (The Scripps Research Institute, La Jolla, CA). Coding sequences of *M. jannaschii* aaRS from plasmid pSup-p*Mj*mutTyrRS-6TRN were amplified by PCR using primers Syn-Nde F (5' – TTA*CATATG*GACGAATTTGAAATGATAAAGAGA –3') and Syn-Bam R (5' – C*GGATCC*TTATAATCTCTTTCTAATTGGCTCTAAAA –3'). The *M. jannaschii* aaRS cDNA copies were cloned directly into a pET-15b vector downstream the T7 promoter, RBS and His-tag regions for efficient expression and purification. Plasmid pET-*Mj*mutTyrRS encoding mutated aaRS was transformed into *E. coli* BL21 (DE3) cells (Invitrogen, Grand Island, NY). All vector constructs were confirmed by DNA sequencing at the sequencing facility of the National Institute for Biotechnology in the Negev (NIBN), Ben-Gurion University of the Negev (BGU).

### Preparation of *Mj*tRNA_CUA_, tRNA_CUA_^Opt^

cDNA copies of all the RNA molecules under regulation of the T7 promoter were obtained by annealing the following two synthetic oligonucleotides: *Mj*tRNA_CUA_ Forward 5'– TAATACGACTCACTATACCGGCGGTAGTTCAGCAGGGCAGAACGGCGGACTCTAAATCCGCATGGCGCTGGTTCAAATCCGGCCCGCCGGACCA–3' and *Mj*tRNA_CUA_ Reverse 5'– TGGTCCGGCGGGCCGGATTTGAACCAGCGCCATGCGGATTTAGAGTCCGCCGTTCTGCCCTGCTGAACTACCGCCGGTATAGTGAGTCGTATTA –3'; and tRNA_CUA_^Opt^ Forward 5'–TAATACGACTCACTATACCGGCGGTAGTTCAGCAGGGCAGAACGGCGGACTCTAAATCCGCATGGCAGGGGTTCAAATCCCCTCCGCCGGACCA–3' and tRNA_CUA_^Opt^ Reverse 5'–TGGTCCGGCGGAGGGGATTTGAACCCCTGCCATGCGGATTTAGAGTCCGCCGTTCTGCCCTGCTGAACTACCGCCGGTATAGTGAGTCGTATTA–3'. Obtained products were used for *in vitro* RNA transcription by TranscriptAid High Yield Transcription Kit (Fermentas, Vilnius, Lithuania). Purified RNA molecules were heated to 90°C for 2 minutes, placed on ice for 2 minutes, and folded by addition of RNA Structuring Buffer (10 mM Tris-HCl, pH 7, 0.1 M KCl, 10 mM MgCl) and kept at 37°C for 20 minutes.

### Preparation of *M. jannaschii* Tyrosyl-tRNA Synthetase and its Evolved Derivatives

*E. coli* BL21 (DE3) cells transformed with one of the above plasmids were grown to an OD_600_ of 0.5–0.7 in 1 L Luria-Bertani (LB) medium. Isopropyl-ß-D-thiogalactoside (IPTG) was added to a final concentration of 1 mM and the cells were grown for additional 4–5 h at 37°C. Cells were harvested at 8,000 g for 10 min at 4°C. The cell pellet was resuspended in 4 mL of lysis buffer (300 mM NaCl, 10 mM imidazole, 50 mM NaH_2_PO_4_, pH 8.0) per gram of cell paste. A cell lysate was prepared using BugBuster Protein Extraction Reagent (Novagen, Darmstadt, Germany) with addition of benzonase nuclease (Novagen) and Protease Inhibitor Cocktail Set III (Merck, Darmstadt, Germany). The lysate was centrifuged at 16,000 g at 4°C for 30 minutes. The His-tagged synthetase was then purified using Ni-NTA agarose (Qiagen). The Ni-NTA agarose beads were washed twice with wash buffer (300 mM NaCl, 20 mM imidazole, 50 mM NaH_2_PO_4_, pH 8.0) and the protein was eluted with elution buffer (300 mM NaCl, 250 mM imidazole, 50 mM NaH_2_PO_4_, pH 8.0). The eluate was dialyzed against sterile PBS buffer pH 7.4 three times and concentrated using an Amicon Ultra-centrifuge device 10 kDa MWCO (Millipore, Beverly, MA).

### Coupled *in vitro* Transcription/translation Reaction

Cell-free protein expression was performed using the RTS 100 *E. coli* HY Kit (5 PRIME, Hamburg, Germany) at 30°C for 6 h in 10 (for Western Blot) or in 50 µL (for protein purification and MS analysis) of reaction mixture. Expression of GFP with an incorporated UAA was achieved by mixing the RTS 100 *E. coli* HY Kit reaction mixture containing 0.5 µg of modified control vector GFP with purified *M. jannaschii* aaRS derivatives (100–450 µg/mL, final concentration) and orthogonal suppressor either *Mj*tRNA_CUA_ or tRNA_CUA_^Opt^ (480–600 µg/mL) in the absence or presence of the corresponding UAA (1 mM).

### Proteins Quantitative Analysis and Purification

Following UAA incorporation using the cell-free expression system, 5 µL of reaction solution were precipitated with acetone to avoid protein aggregation. The protein pellet was resuspended in an equal volume of LDS loading buffer (Invitrogen). The resulting solution was incubated at 70°C for 10 minutes and resolved by SDS-PAGE. Proteins of interest were visualized by Coomassie staining using SimplyBlue SafeStain (Invitrogen). For Western blot analysis, proteins were transferred to a nitrocellulose membrane using Immuno-Blot PVDF membrane apparatus (Bio-Rad, Hercules, CA). To visualize proteins in the Western blot, we used primary mouse monoclonal IgG directed against His-tag (Santa Cruz Biotechnology, Santa Cruz, CA) and horseradish peroxidase-conjugated goat polyclonal secondary antibodies to mouse IgG1 heavy chain (Abcam, Cambridge, UK). Chemiluminescence was detected with SuperSignal West Pico Chemiluminescent Substrate (Thermo Scientific, Rockford, IL). Absolute quantities of expressed proteins were estimated by purification of WT and mutated GFP by Ni-NTA agarose (Qiagene), concentration by Amicon Ultra-centrifuge device 10 kDa MWCO (Millipore) and resolving by SDS-PAGE. Proteins of interest were visualized by Coomassie staining using SimplyBlue SafeStain (Invitrogen), the desired proteins bands were cut from the gel and eluted using Model 422 Electro-Eluter (Bio-Rad). The concentration of proteins were measured by Implen Nanophotometer (Labfish, Germany). The relative quantity of expressed proteins was analyzed by densitometry using GeneTools software (SynGene, Cambridge, UK). All of the proteins expressed using the RTS 100 *E. coli* HY Kit were purified according to the manufacturer’s manual using Ni-NTA Spin Columns (Qiagen, Hilden, Germany).

### Proteolysis and Mass Spectrometry

The trypsin cleavage sites in WT GFP were predicted by PeptideCutter (http://web.expasy.org/peptide_cutter/). In-gel digestion of purified GFP containing UAA, was performed using Trypsin Gold (Promega, Madison, WI) according to a modified manufacturer’s procedure. Briefly, the gel bands corresponding in size to GFP were finely sliced, washed for 5 minutes with water, 15 minutes with acetonitrile (ACN), and dried in a vacuum centrifuge. After reduction (10 mM dithiothreitol in 100 mM NH_4_HCO_3_) and alkylation (10% iodoacetamide in 100 mM NH_4_HCO_3_), dried gel slices were incubated on ice for 15–30 minutes with Trypsin gold (12.5 ng/µL) in digestion buffer (50 mM NH_4_HCO_3_, 5 mM CaCl_2_). Excess buffer was removed and 20–30 µL of digestion buffer without Trypsin Gold was added to the gel slices followed by incubation at 37°C overnight. Peptides were extracted from the gel slices by washing once with 25 mM NH_4_HCO_3_, ACN and 1% formic acid (FA). The samples were then dried in a vacuum centrifuge. The extracted peptides were purified and concentrated using ZipTip pipette tips (Millipore), following the manufacturer’s instruction.

ESI-MS/MS analysis was performed using reverse phase nano-LC (Agilent Technology) connected directly to the LTQ XL Orbitrap EDT mass spectrometer (Thermo Electron, Wien, Austria) at the Analytical Research Services & Instrumentation Unit, BGU. The peptides were eluted with an increasing ACN gradient (Solvent A, 0.1% FA, 5% ACN; Solvent B, 0.1% FA, 80% ACN) over a period of 70 min. MS/MS spectra were acquired in a data-dependent fashion. Instrument control was performed using the Xcalibur software package (Thermo Electron).

Theoretical monoisotopic masses for the peptides generated by trypsin digestion of WT GFP were predicted with PeptideMass (http://web.expasy.org/peptide_mass/), while fragmentation of the FSVSGEGEGDATYGK peptide and theoretical molecular masses of the peptide species were calculated with the MS-Product software at the ProteinProspector web service (http://prospector.ucsf.edu/prospector/cgi-bin/msform.cgi?form=msproduct). Theoretical molecular masses for peptides containing UAA were adjusted manually.

## Results

### Site-specific Incorporation of Tyrosine into GFP in Response to a UAG-stop Codon in a Cell-free Expression System

**Figure 2:**
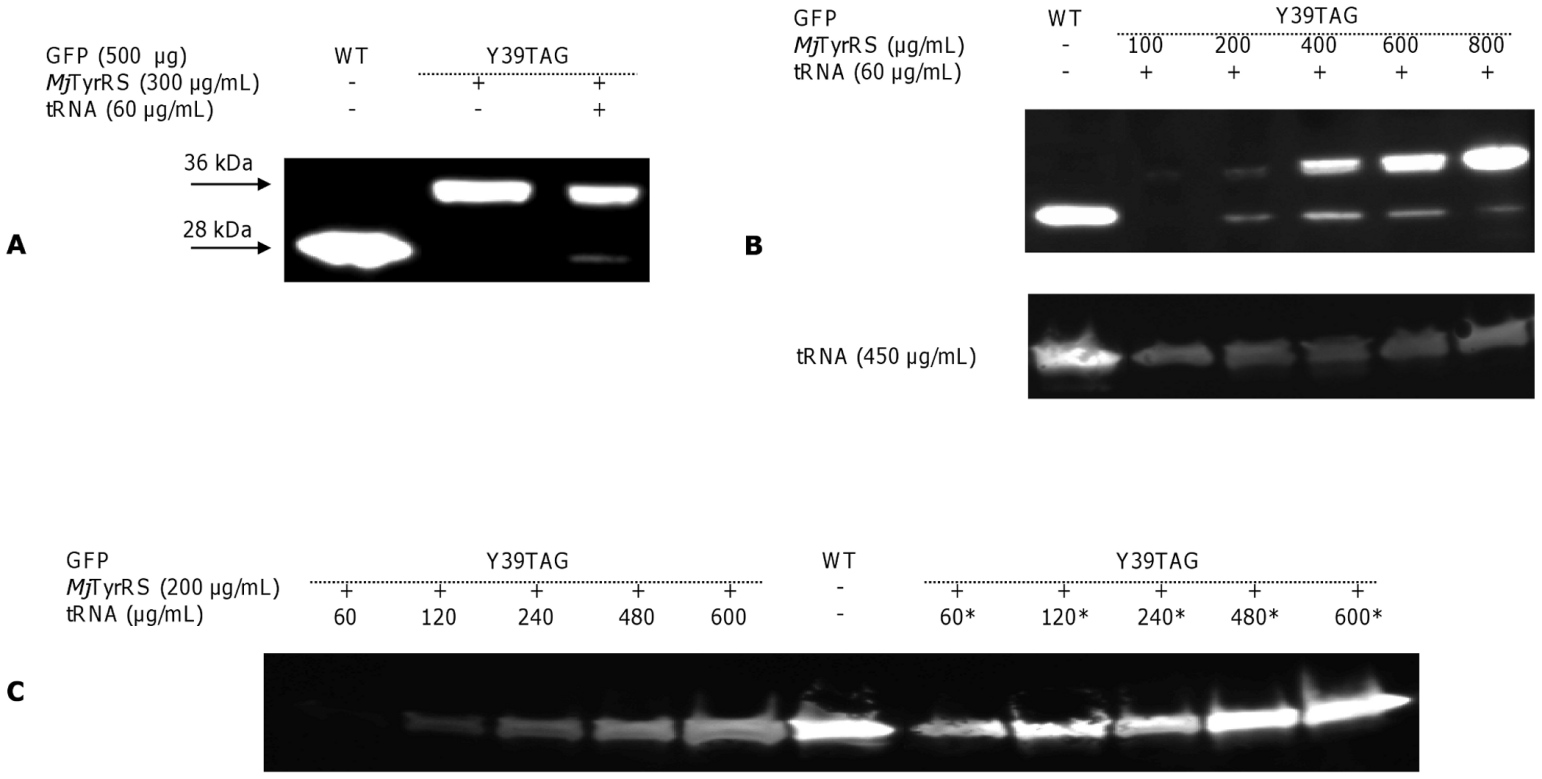
Western Blot of WT GFP and GFP Y39TAG mutant expression in a cell-free translation system. Synthesis of WT GFP and the GFP Y39TAG mutant was performed using the RTS *E. coli* HY Kit, to which the corresponding plasmid (500 µg/mL), purified *Mj*TyrRS and cognate suppressor *Mj*tRNA_CUA_ (tRNA) or T-stem modified tRNA_CUA_^Opt^ (denoted as *) were added. (A) Expression of WT GFP and the GFP Y39TAG mutant in the presence of *Mj*TyrRS (300 µg/mL) and synthetic *Mj*tRNA_CUA_ (60 µg/mL). The band at 28 kDa corresponds to full-length GFP. (B) Western blot analysis demonstrates enhanced GFP Y39TAG protein expression as a function of increased *Mj*TyrRS concentrations in a cell-free reaction medium supplied with *Mj*tRNA_CUA_ (60 µg/mL – top panel and 450 µg/mL – bottom panel). (**C**) Dependence of GFP Y39TAG yield on the type and concentration of nonsense suppressor, as visualized by Western blot.

To incorporate tyrosine in response to TAG stop codon we expressed plasmid GFP Y39TAG obtained by site-directed mutagenesis in the RTS 100 *E. coli* HY Kit mixture supplied with external components, purified *Mj*TyrRS and a suppressor *Mj*tRNA_CUA_; we employed GFP, encoded by a control vector of the kit, as a reporter protein. Western blot with anti His-antibodies enables to visualize full-length and not truncated GFP at a size of 28 kDa, as well as *Mj*TyrRS of 36 kDa (Fig. 2A). The addition of purified *Mj*TyrRS and synthetic *Mj*tRNA_CUA_ to the reaction mixture permitted site-specific incorporation of tyrosine in response to the stop codon at GFP Y39TAG-mutated proteins, while no bands corresponding in size to GFP were detected in the reaction mixture supplied only with *Mj*TyrRS, indicating orthogonality of *M. jannaschii* synthetase to endogenous tRNA molecules. Since estimated band intensity corresponding to GFP Y39TAG did not exceed 10% of the WT expression level, we further adjusted *Mj*TyrRS and *Mj*tRNA_CUA_ concentrations in the reaction mixture. The synthetase concentration required for maximal suppression efficiency was found to vary widely and depended on the concentration of *Mj*tRNA_CUA_ (Fig. 2B). At a *Mj*TyrRS concentration equal to 400–450 µg/mL and in the presence of 60 µg/mL *Mj*tRNA_CUA_, the expression level of Y39TAG GFP reached the maximum possible under these conditions and was roughly 20% of WT expression level, while in the presence of 450 µg/mL *Mj*tRNA_CUA_, the maximal expression level reached 35% of WT expression level and required only 100 µg/mL of *Mj*TyrRS. To determine whether the suppressor tRNA structure is limiting, we sought another suppressor. We then adjusted the concentration of both *Mj*tRNA_CUA_ and T-stem-modified tRNA_CUA_^Opt^ for optimal tyrosine incorporation. Using both the original and alternate suppressor, the expression of full-length GFP was demonstrated to depend greatly on the nonsense suppressor concentration (Fig. 2C). A maximum yield of Y39TAG GFP constituting 55% and 115% of the WT expression level was achieved at *Mj*tRNA_CUA_ concentration of 600 µg/mL and of a tRNA_CUA_^Opt^ concentration of 480 µg/mL, respectively. This experiment revealed that the nature of suppressor tRNA significantly affects the efficiency of TAG nonsense suppression in recombinant proteins, while application of tRNA_CUA_^Opt^ allows one to reach at least similar protein yields as that of WT protein.

### The Effect of the Nucleotide following the Stop Codon on the Expression of UAA-containing Proteins

**Figure 3:**
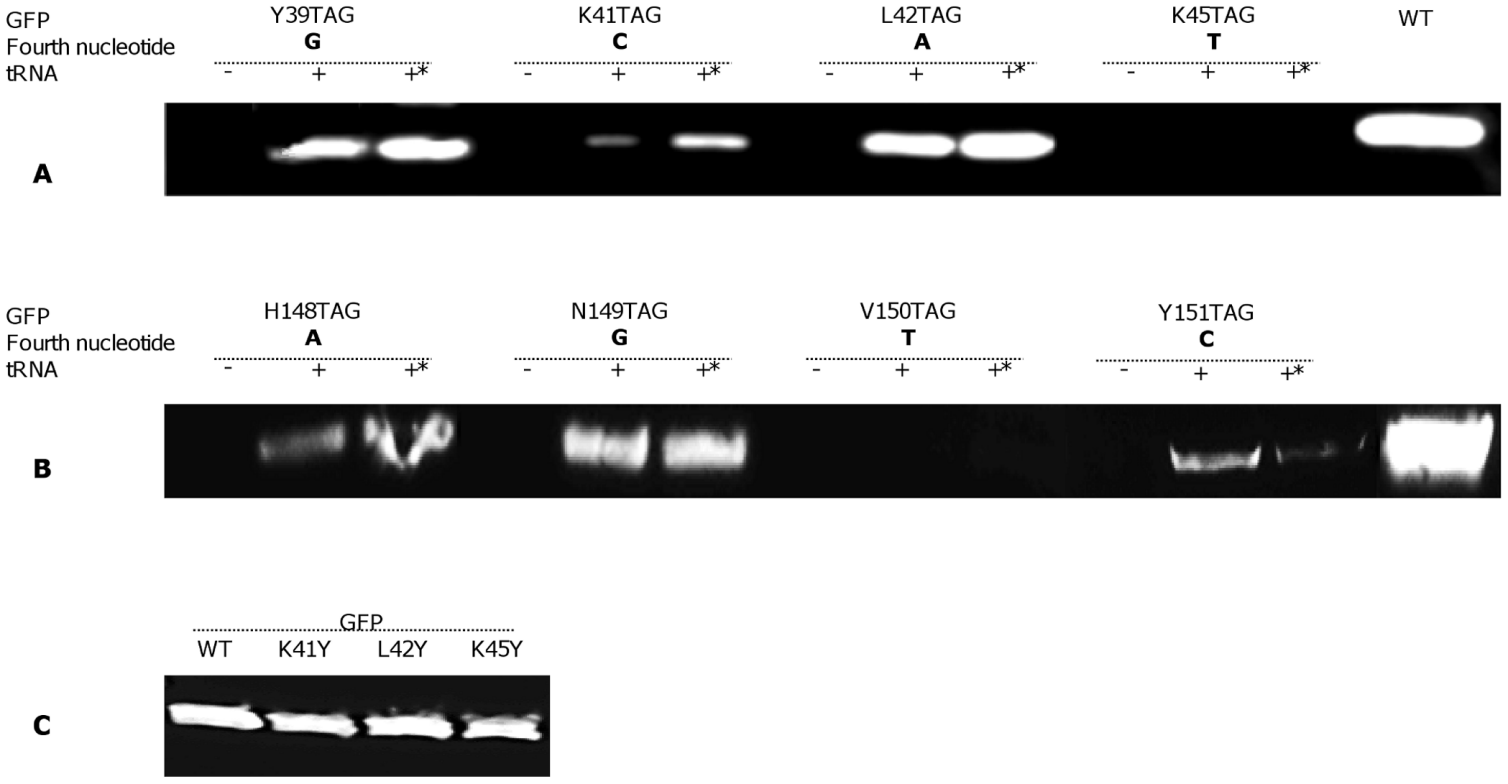
Cell-free expression of WT GFP and tyrosine-incorporating mutant GFP, as visualized by Western blot. (A and B) Co-translational incorporation of tyrosine at different positions in response to the amber stop codon was achieved by adding purified *Mj*TyrRS (200 µg/mL) and two types of suppressor tRNA (480 µg/mL) to the reaction mixture (tRNA denotes synthetic *Mj*tRNA_CUA_, *– tRNA_CUA_^Opt^). (C) Western blot visualization of the expression level of GFP WT and tyrosine-substituted proteins.

As it is mentioned above, the TAG stop codon specifying the desired position for UAA incorporation into a recombinant protein could be recognized by either RF1 or by the cognate suppressor tRNA. It has been shown that the identity of the nucleotide following nonsense codon impinge on the selection rate of RF1 [12], i.e. a low rate of stop signal recognition by RF1 means that mRNA interaction with near-cognate aminoacyl-tRNA or frame-shifting occurs faster than does RF1 binding to the ribosomal A-site [12]. Based on the literature screened [17], [19], [22], [25], [26], we suggested that the forth nucleotide in tetra-nucleotide stop signal could affect the rate of nonsense suppression [12], [13]. To verify this notion, we selected several amino acids in two different GFP β-sheets and their adjacent loop that were substituted to TAG, the location was selected in a way that afforded that the immediate nucleotide downstream from the stop codon differed from one location to another. Thus, we constructed plasmids encoding GFP Y39TAG, K41TAG, L42TAG and K45TAG mutants, where the amber codon was followed by a guanine (G), a cytosine (C), an adenine (A) and a thymine (T), respectively; and GFP H148TAG, N149TAG, V150TAG and Y151TAG were stop codon was followed by A, G, T and C, respectively. All these constructs were expressed using the cell-free expression kit supplemented by *Mj*TyrRS (150 µg/mL) in the absence or presence of either *Mj*tRNA_CUA_ or tRNA_CUA_^Opt^ (480 µg/mL). Western blot analysis (Fig. 3A, 3B) revealed that the expression level of full-length GFP depended on the specific nucleotide following TAG stop codon in the cell-free protein translation system based on an *E. coli* lysate. The fourth nucleotide hierarchy for efficient suppression was demonstrated to be A≈G>C>T for both variants of nonsense suppressor tRNAs. A literature screen revealed that *E. coli*-based cell-free protein synthesis had been successfully employed for UAA incorporation into proteins when the TAG stop codon was followed by G [22], [26], A [19], [25], and C [22]. To the best of our knowledge, no expression of protein containing UAA has been reported when T followed the stop codon. To demonstrate that indeed the “following base” and not position change in the protein affects the rate of suppression efficiency, we have substituted K41Y, L42Y and K45Y to tyrosine and tested GFP expression level for these mutants relative to the WT. It should be noted that, the fourth nucleotides after the tyrosine codon remained as in the native sequence: G after the codon in the native protein GFP39Y, C after GFP K41Y, A after GFP L42Y and T after K45Y. Western blot analysis of these control mutants revealed that expression levels did not differ from those of WT and from one another (Fig. 3C), verifying that indeed the effects that we have observed imply context dependence.

### Genetic Incorporation of UAA in Response to the Amber Stop Codon

**Figure 4:**
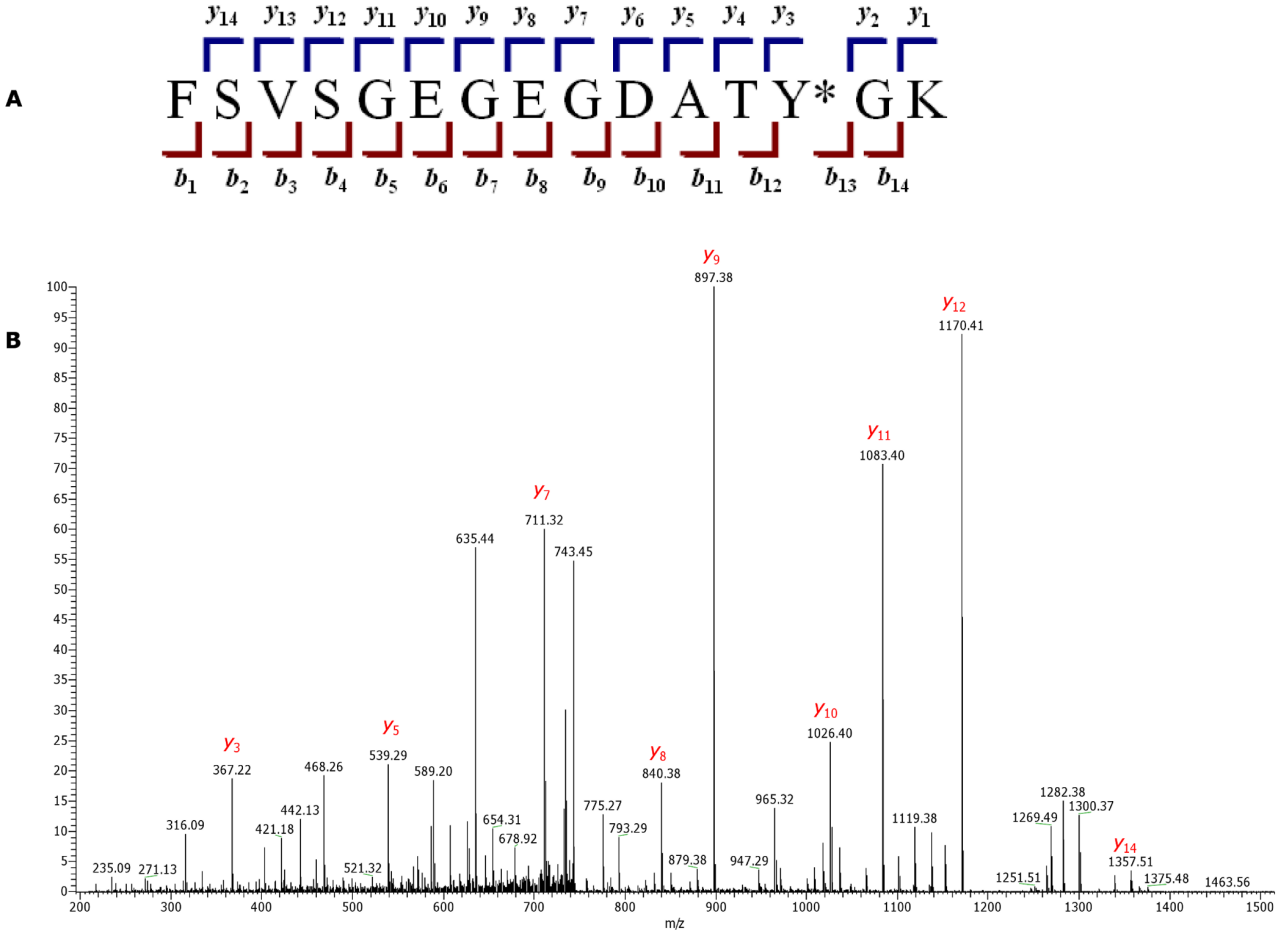
Site-specific Bpa incorporation into GFP in a cell-free expression system. (A) Western blot visualization of WT GFP and Bpa-incorporating GFP Y39TAG. The synthesis of GFP was performed using an *in vitro* translation kit. Co-translational incorporation of Bpa was achieved by addition of *M. jannaschii* BpaRS (100 µg/mL), different types of suppressor tRNA (480 µg/mL) and Bpa (1 mM) to the reaction medium. tRNA denotes synthetic *Mj*tRNA_CUA_, Opt – tRNA_CUA_^Opt^. (B) Annotated MS/MS spectrum of the FSVSGEGEGDATY*GK peptide from GFP Y39Bpa. The mass shift of 88 Da between Bpa in GFP Y39Bpa and tyrosine in WT GFP is clearly observed. For clarity, only the most abundant “y” ions are assigned.

**Figure 5:**
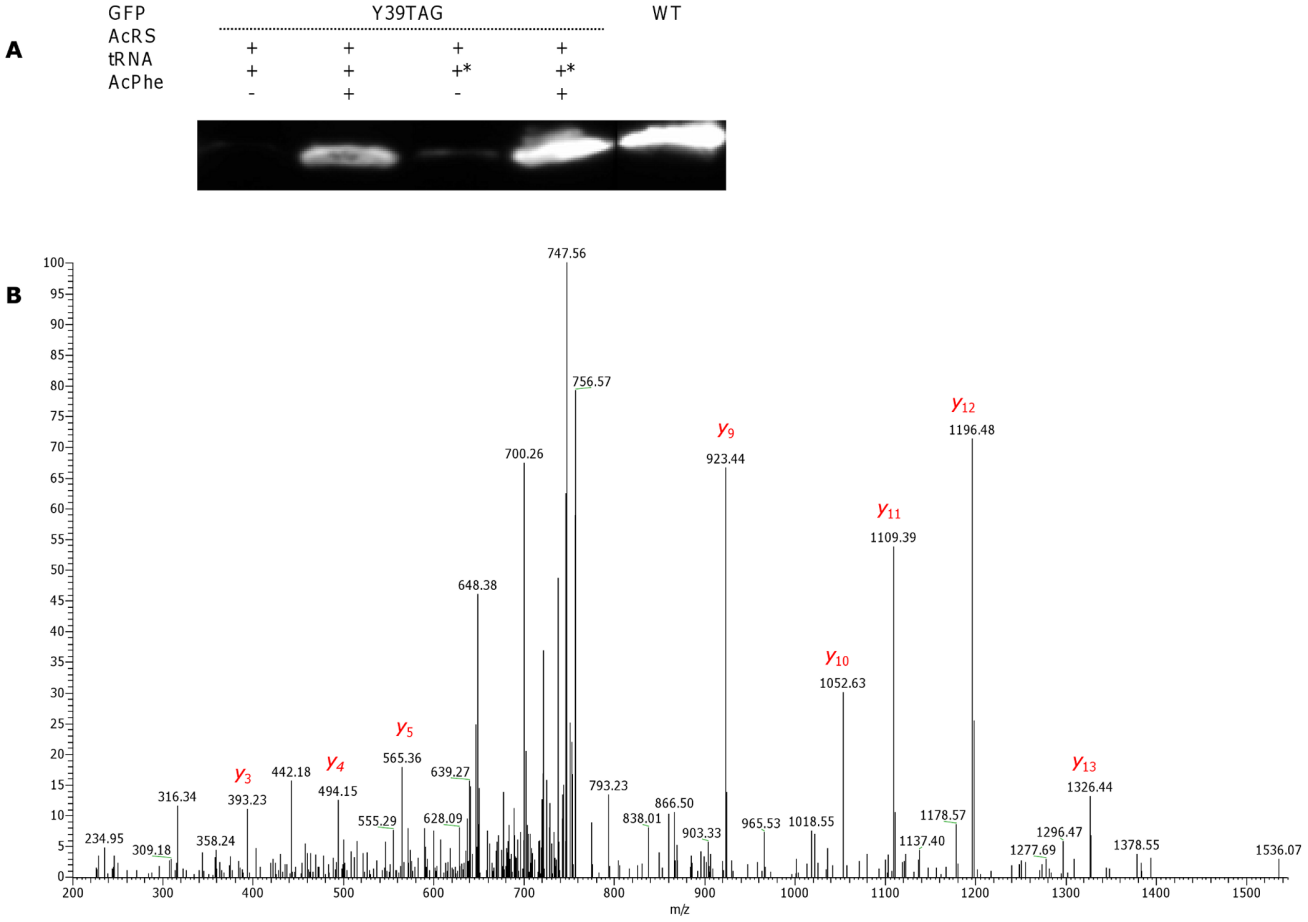
Site-specific incorporation of pIPhe into GFP in a cell-free expression system. (A) Western blot visualization of WT GFP and pIPhe-incorporated GFP Y39TAG. Co-translational incorporation of pIPhe was achieved by addition of *M. jannaschii* IPheRS (100 µg/mL), different types of suppressor tRNA (480 µg/mL), and pIPhe (1 mM) to the reaction medium. tRNA denotes synthetic *Mj*tRNA_CUA_, Opt – tRNA_CUA_^Opt^. (B) Annotated MS/MS spectrum of the FSVSGEGEGDATY*GK peptide from GFP Y39IPhe. The mass shift between pIPhe and tyrosine was calculated to be 109.9 Da, corresponding to the observed “y” ions.

**Figure 6:**
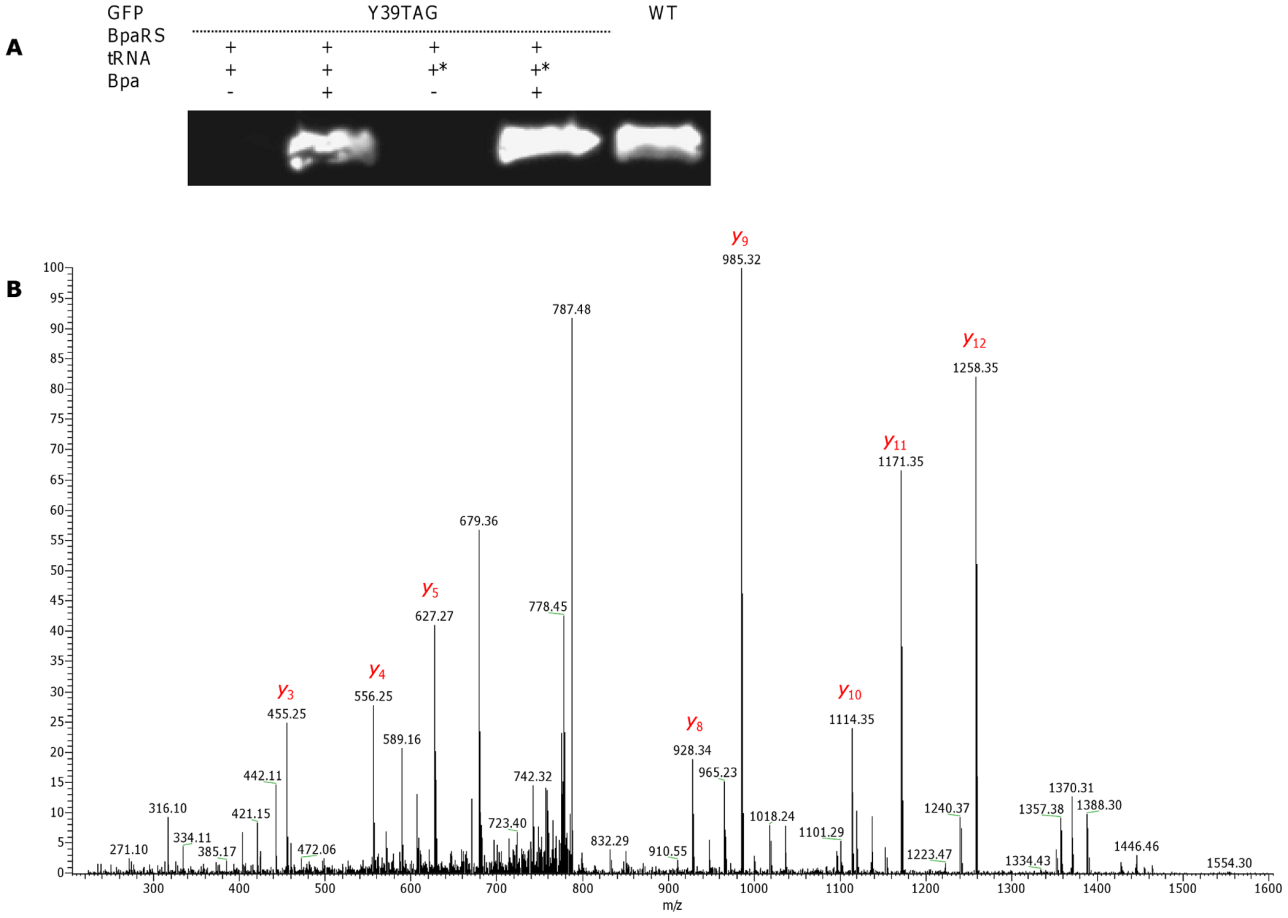
Site-specific pAcPhe incorporation into GFP in a cell-free expression system. (A) Western blot visualization of WT GFP and pAcPhe-incorporating GFP Y39TAG. The synthesis of GFP was performed using an *in vitro* translation kit. Co-translational incorporation of pAcPhe was achieved upon addition of the corresponding plasmid (500 µg/mL), *M. jannaschii* pAcPheRS (100 µg/mL), different types of suppressor tRNA (480 µg/mL), and pAcPhe (1 mM) to the reaction medium. tRNA denotes synthetic *Mj*tRNA_CUA_, Opt – tRNA_CUA_^Opt^. (B) Annotated average MS/MS spectrum of peptides with *m/z* 765.84 corresponding to pAcPhe-incorporated FSVSGEGEGDATY*GK peptide of GFP Y39TAG. The Y* ion corresponding to pAcPhe (calculated *m/z*, 393.24; observed *m/z*, 393.23) can be easily detected. The distinguishing mass shift of 26 Da can be observed for most abundant “y” ions. For clarity, only the most abundant “y” ions are assigned.

To test the generality of the developed platform, we examined its ability to incorporate diverse UAAs at position 39 of GFP in response to the TAG stop codon, applying both types of suppressor tRNAs and three variants of *Mj*TyrRS derivatives. The three evolved variants of *M. jannaschii* aaRS, i.e. AcRS [27], BpaRS [28] and IPheRS [29], were tested for the ability to suppress the amber stop codon in GFP Y39TAG mutants together with either *Mj*tRNA_CUA_ or tRNA_CUA_^Opt^ in the absence or presence of their cognate UAA in a cell-free translation system. The expression of full-length GFP Y39TAG was shown (Fig. 4A and 5A) to depend on the presence of pBpa and pIPhe. GFP expression was not detected in the absence of pBpa and pIPhe. Although AcRS has been widely used for site-specific protein labeling *in vivo* [17], [30], [31], its application in cell-free reaction medium led to background suppression in the absence of pAcPhe (Fig. 6A). The reason for background suppression *in vivo* is from mis-acylation of the suppressor tRNA molecules by the evolved synthetase with an endogenous amino acid, such as tyrosine or phenylalanine, in the rich media [17]. The overall level of background suppression was estimated to be less than 2 and 4.5% of GFP WT expression level for *Mj*tRNA_CUA_ and tRNA_CUA_^Opt^, respectively; however, since the main disadvantage of using previously reported eukaryotic-based cell-free systems for UAA incorporation was a high degree of mis-acylation with endogenous amino acids [21], site-specifically modified GFP Y39TAG were further characterized by mass spectrometry (MS).

The relative yields of GFP Y39TAG protein with site-specifically incorporated pIPhe and pAcPhe were measured to be 45–52% for the reactions utilizing *Mj*tRNA_CUA_, and an approximately 85% for tRNA_CUA_^Opt^ (Fig. 5A and 6A). The highest efficiency of UAA incorporation was obtained by applying BpaRS to the cell-free reaction mixture; the expression level of GFP Y39-mutated proteins comprised an approximately 80 and 120% of GFP WT level for *Mj*tRNA_CUA_ and tRNA_CUA_^Opt^, respectively (Fig. 4A).

### Mass Spectrometry Analysis to Determine Specificity and Fidelity of Incorporation

**Figure 7:**
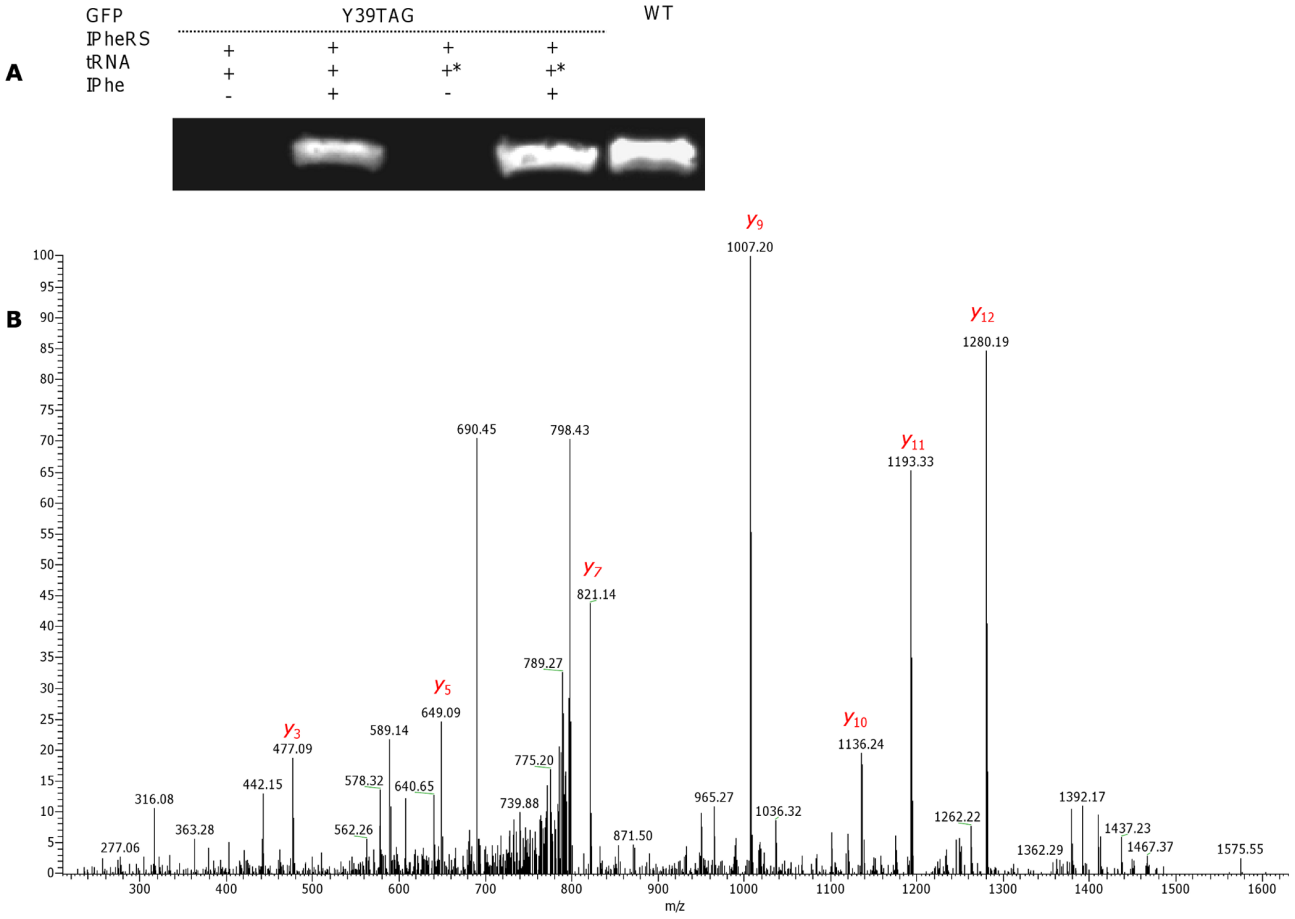
Annotated tandem MS spectra of the FSVSGEGEGDATY*GK peptide from WT GFP. (A) “y”- and “b”-type ions generated during fragmentation of the FSVSGEGEGDATY*GK peptide. Y* denotes tyrosine in WT GFP or UAA in the GFP Y39TAG mutants. (B) MS/MS spectrum of FSVSGEGEGDATY*GK from WT GFP.

To examine the specificity and fidelity of *M. jannaschii* aaRS derivatives, WT GFP and GFP Y39TAG were expressed using a cell-free translation system, purified and separated by SDS-PAGE. The bands corresponding in size to GFP, as visualized by Coomassie staining, were excised, trypsin-digested and analyzed with a LTQ XL Orbitrap EDT mass spectrometer. The peptide of interest was predicted by the PeptideCutter software to be FSVSGEGEGDATY*GK, where Y* denotes either tyrosine in WT GFP or UAA in GFP Y39TAG. The monoisotopic mass calculated by PeptideMass software for this peptide generated by trypsin digest of the WT protein was 1503.6597 Da; the mass of the same peptide from GFP Y39TAG was adjusted manually (depending on the incorporated UAA mass). MS analysis of WT GFP identified the desired peptide, showing a doubly charged ion [M^+2^] peak of *m/z* 752.829, in a good agreement with the calculated mass. Peaks observed in the average MS/MS spectrum (Fig. 7) were also in a good agreement with predicted masses (calculated with the ProteinProspector MS-Product software) of “y” and “b” ions generated by FSVSGEGEGDATY*GK peptide fragmentation (Table S1). MS analysis of GFP Y39TAG revealed peaks with doubly charged ion masses of 765.84 (calculated mass, 765.85), 807.79 (calculated mass, 807.78) and 796.85 (calculated mass, 796.88) for FSVSGEGEGDATY*GK peptides containing pAcPhe, pBpa and pIPhe, respectively. Although Western blot revealed some degree of background suppression of amber stop codon of GFP Y39TAG obtained by using AcRS in the absence of pAcPhe, no peptide signals corresponding to GFP sequence containing tyrosine or phenylalanine at position 39 were detected, indicating that similarly to the observed *in vivo* process [17], [27] mis-acylation of suppressor tRNA by near-cognate endogenous amino acids was inhibited by addition of cognate UAA. Addition of higher concentrations of the UAA to the reaction, eliminate the competition fully, as no molecules with *m/z* equivalent to trypsin digested WT GFP were found for proteins obtained by addition of UAAs to the cell-free reaction. Moreover, no peptides containing canonical amino acids from near-cognate suppression of stop codon were detected, confirming good selectivity and fidelity of the analyzed *M. jannaschii* aaRSs. MS/MS analysis of GFP Y39TAG peptides (Fig. 4B, 5B and 6B) demonstrated a characteristic mass shift of 88.11, 109.9 and 26.04 Da relative to WT GFP, values that exactly match the differences between tyrosine and pBpa, pIPhe and pAcPhe, respectively. Mass shifts were also detected for peaks corresponding to “y” ions (from y_3_ to y_14_), as well as for b_13_ ion, indicating the site of UAAs incorporation to be position 39 of GFP. In general, we did not detect any differences in the GFP Y39TAG proteins obtained by utilization of either *Mj*tRNA_CUA_ or tRNA_CUA_^Opt^, apart from the different protein yields.

## Discussion

Since it has been shown that protein synthesis is a ribosome-mediated process that does not require cell integrity, cell-free protein expression systems have been used for a variety of purposes, including site-specific UAA incorporation into recombinant proteins [21], [26]. In our efforts to develop an *E. coli*-based cell-free protein expression system to produce high yields of UAA containing recombinant proteins, we employed the most established *M. janaschii* orthogonal synthetases and nonsense suppressor molecules. In contrast to previously described cell-free expression systems adapted for protein synthesis with encoded UAAs, we employed purified *Mj*TyrRS and its derivatives along with suppressor tRNA as additional components of defined reaction mixture allowing for control of the protein synthesis environment. In this manner, we were able to produce GFP Y39TAG with tyrosine with an absolute yield of 270 µg/mL and a suppression efficiency of 55% by adjusting *Mj*TyrRS and *Mj*tRNA_CUA_ concentrations. Although suppressor tRNA concentration is the major factor limiting production of proteins containing UAAs, further augmentation of the proportion of *Mj*tRNA_CUA_ in the reaction medium (exceeding a final concentration of 600 µg/mL) led to the inhibition of recombinant protein biosynthesis, presumably because of translational apparatus overloading with non-endogenous elements. Replacement of the orthogonal *Mj*tRNA_CUA_ suppressor by T-stem-modified tRNA_CUA_^Opt^ optimized for efficient recognition and binding by *E. coli* EF-Tu factor resulted in further enhancement of recombinant protein yields, which were estimated to be 120% of WT GFP expression. It was previously shown that the degree of improvement in suppression efficiency of evolved tRNA_CUA_^Opt^ varied, depending on the specific aaRS and cognate UAA used [17], [24]. We have also observed the ability of modified tRNA_CUA_^Opt^ to suppress amber stop codon *in vitro* with different efficiencies, depending on the UAA being incorporated. The application of optimized suppressors in a cell-free reaction medium for most aaRSs resulted in an enhanced protein yield, ranging from 85 to 110% of WT levels. Still, we cannot exclude the possibility that some of the evolved *Mj*TyrRSs could have lower affinity to such a suppressor. For both tRNA molecules, the high fidelity of *M. jannaschii* aaRS derivatives in the cell-free expression system was confirmed by mass spectrometry.

The efficiency of UAAs incorporation in response to a stop codon is known to depend on the position of the mutation site and the nature of the recombinant protein, in particular the encoded amino acids and corresponding codon surrounding the amber codon. Although this phenomenon is usually taken into consideration when designing experiments, the precise reason and mechanism leading to this observation was not reported. It is well known that the identity the of nucleotide following stop codon influence the efficiency of translation termination. Depending on the nucleotide downstream the certain stop codon, the decoding site of 16S ribosomal RNA (rRNA) favors either translational termination by binding to the RF-factors, “read-through” of stop codon by augmentation of near-cognate aminoacyl-tRNA binding or a “frame-shift” [12], [32]. We hypothesized that the effectiveness of suppressor tRNA binding to its cognate nonsense codon would also depend on the following nucleotide. The expression of GFP Y39TAG, K41TAG, L42TAG and K45TAG mutants (where the fourth nucleotide was G, C, A, and T, respectively), and of GFP H148TAG, N149TAG, V150TAG and Y151TAG (where the fourth nucleotide was – A, G, T and C, respectively) demonstrated that the identity of the base following the amber codon determined the efficiency of UAA-charged suppressor *Mj*tRNA_CUA_ or tRNA_CUA_^Opt^ interaction with UAG stop codon and, as a result, overall protein yields. According to our study, the strength of UAG stop codon selection and interaction was predicted by the fourth base hierarchy to be A≈G>C>T for both *Mj*tRNA_CUA_ and tRNA_CUA_^Opt^. The purines as the forth nucleotide improve the termination in the presence of RF1, however, we suggest that in the cell-free reaction medium prepared by us concentration of suppressor tRNA exceeds the concentration of RF1 many times rendering RF1 unavailable to the ribosomes; under these conditions, a purine nucleotide downstream the stop codon would augment UAG interaction with its cognate suppressor tRNA over the possibilities of mis-acylation or frame-shift. Analysis of the literature failed to find an example of the incorporation of any UAA in response to the amber stop codon followed by a T. Instead, the base following the stop codon was either G or A for the majority of UAA-containing proteins produced *in vitro* and *in vivo* that we have screened. These findings indirectly confirm our observation concerning the effect of the nucleotide downstream of the stop codon on the efficiency of UAA incorporation. Nonetheless, the actual mechanism by which the fourth base modulates the efficiency of stop codon suppression has yet to be revealed. In addition we would like to stress that a statistical analysis was not performed, hence we base our conclusions only on experimental evidence in our study combined with a literature screen.

It is also important to note that all of the reported protein yields are not absolute quantities and are reported as relative values and as percent of the WT expression levels, which were set to 100% in this study.

### Conclusions

The cell-free translation system, modified as reported here, to genetically encode proteins containing UAAs resulted in increased amounts of recombinant proteins with very good fidelity. Concentrations of added UAAs with cognate RSs that have shown lower fidelity, can be tuned in this system in a controled manner, thus eliminating possible competition of incorporation of natural amino acids. Compettition of supressor tRNA with RF1 can be reduced significantly by using controled and higher concentrations of suppressor tRNA, thus affording higher supression efficiencies. The ability to control the concentrations of the different orthogonal components in this system afford reduced competition from natural components in the translational machinery.

The major advantage of the methodology reported here is its generality. Due to the availability of commercial cell-free translation systems with variety of modifications, it is possible to produce both prokaryotic and eukaryotic UAA-encoded proteins. The nature of the *in vitro* approach enables one to incorporate UAAs into nascent polypeptides that are not available for living organisms, provided that the right aaRS is available. It is also our belief that through this approach, more than one UAA could be incorporated into a protein with only a small loss in protein yield.

## Supporting Information

Table S1 Calculation of “y” and “b” ions of the FSVSGEGEGDATY*GK fragment (Y* denotes either tyrosine in WT GFP or UAA in the GFP Y39TAG mutants). Masses for the WT GFP-derived FSVSGEGEGDATY*GK peptide fragmentation were predicted by the MS-Product software of the ProteinProspector web service, while masses for GFP Y39TAG mutants were adjusted manually.
(DOC)
