# Supplementary material for: Enhanced Yield of Recombinant Proteins with Site-Specifically Incorporated Unnatural Amino Acids Using a Cell-Free Expression System
Source: PLoS One. 2013 Jul 2;8(7):e68363. doi: 10.1371/journal.pone.0068363 (PMC3699557; doi:10.1371/journal.pone.0068363)
Supplement: Table S1 — Calculation of “y” and “b” ions of the FSVSGEGEGDATY*GK fragment (Y* denotes either tyrosine in WT GFP or UAA in the GFP Y39TAG mutants). Masses for the WT GFP-derived FSVSGEGEGDATY*GK peptide fragmentation were predicted by the MS-Product software of the ProteinProspector web service, while masses for GFP Y39TAG mutants were adjusted manually. (DOC) [file pone.0068363.s001.doc]

|  |  | [M^+^] b ions | | | |
| --- | --- | --- | --- | --- | --- |
| Residue | # | GFP WT | GFP  Y39TAG  with pAcPhe | GFP  Y39TAG  with pBpa | GFP  Y39TAG  with pIPhe |
| FS | 2 | 235.11 | - - - | - - - | - - - |
| FSV | 3 | 334.18 | - - - | - - - | - - - |
| FSVS | 4 | 421.21 | - - - | - - - | - - - |
| FSVSG | 5 | 478.23 | - - - | - - - | - - - |
| FSVSGE | 6 | 607.27 | - - - | - - - | - - - |
| FSVSGEG | 7 | 664.29 | - - - | - - - | - - - |
| FSVSGEGE | 8 | 793.34 | - - - | - - - | - - - |
| FSVSGEGEG | 9 | 850.36 | - - - | - - - | - - - |
| FSVSGEGEGD | 10 | 965.38 | - - - | - - - | - - - |
| FSVSGEGEGDA | 11 | 1036.42 | - - - | - - - | - - - |
| FSVSGEGEGDAT | 12 | 1137.47 | - - - | - - - | - - - |
| FSVSGEGEGDATY* | 13 | 1300.53 | 1326.57 | 1388.64 | 1433.43 |
| FSVSGEGEGDATY*G | 14 | 1357.55 | 1383.59 | 1445.66 | 1490.45 |
|  | | | | | |
|  |  | [M^+^] y ions | | | |
| Residue | # | GFP WT | GFP  Y39TAG  with pAcPhe | GFP  Y39TAG  with pBpa | GFP  Y39TAG  with pIPhe |
| K | 1 | 147.11 | - - - | - - - | - - - |
| KG | 2 | 204.13 | - - - | - - - | - - - |
| KGY* | 3 | 367.20 | 393.24 | 455.31 | 477.10 |
| KGY*T | 4 | 468.25 | 494.29 | 556.36 | 578.15 |
| KGY*TA | 5 | 539.28 | 565.32 | 627.39 | 649.18 |
| KGY*TAD | 6 | 654.31 | 680.35 | 742.42 | 764.21 |
| KGY*TADG | 7 | 711.33 | 737.37 | 799.44 | 821.23 |
| KGY*TADGE | 8 | 840.37 | 866.41 | 928.48 | 950.27 |
| KGY*TADGEG | 9 | 897.39 | 923.43 | 985.50 | 1007.29 |
| KGY*TADGEGE | 10 | 1026.44 | 1052.48 | 1114.55 | 1136.34 |
| KGY*TADGEGEG | 11 | 1083.46 | 1109.5 | 1171.57 | 1193.36 |
| KGY*TADGEGEGS | 12 | 1170.49 | 1196.53 | 1258.60 | 1280.39 |
| KGY*TADGEGEGSV | 13 | 1269.56 | 1295.6 | 1357.67 | 1379.46 |
| KGY*TADGEGEGSVS | 14 | 1356.59 | 1382.63 | 1444.70 | 1466.49 |
